# Genome-Wide Identification and Drought Stress-Responsive Expression Profiling of the FAD Gene Family in Pear

**DOI:** 10.3390/life15081279

**Published:** 2025-08-12

**Authors:** Ziyi Zhang, Zhikun Li, Yan Zeng, Yutong Zhu, Wenxuan Chu, Ruigang Wu, Qingjiang Wang

**Affiliations:** School of Landscape and Ecological Engineering, Hebei University of Engineering, Handan 056038, China; y2233451088@163.com (Z.Z.); lizhikun741531903@163.com (Z.L.); 18854861590@163.com (Y.Z.); 18265025836@163.com (Y.Z.);

**Keywords:** pear (*Pyrus bretschneideri*), fatty acid desaturase, FAD gene family, bioinformatics, expression analysis

## Abstract

Fatty acid desaturase (FAD) is a rate-limiting enzyme catalyzing the biosynthesis of unsaturated fatty acids (UFAs) and participates in key physiological processes such as plant growth and development, fruit ripening, and stress responses by regulating membrane lipid composition. Using pear genome data, this study systematically identified *FAD* gene family members through bioinformatic analysis and characterized their drought-responsive expression patterns. Results revealed that 34 FAD family members were identified in pear, unevenly distributed across 12 chromosomes and classified into six subfamilies. Members within the same subfamily exhibited similar conserved domains and gene structures. Promoter element analysis demonstrated that pear FAD promoters contain cis-acting elements associated with plant growth and development, hormone responses, and abiotic stress responses. qRT-PCR expression profiling showed that *PbrFAD23* and *PbrFAD30* were significantly upregulated during the early stages of drought stress, followed by suppressed expression levels, suggesting their potential crucial regulatory roles in the initial drought response. Genome-wide identification of 34 PbrFAD family members highlighted that *PbrFAD23* and *PbrFAD30*, with marked upregulation under early drought stress, exhibit prominent drought responsiveness. This study provides valuable resistance gene resources for molecular breeding of stress-tolerant pear varieties and establishes a theoretical foundation for functional characterization of key drought-resistant candidate genes in pear.

## 1. Introduction

Fatty acids (FAs), as crucial hydrophobic components within cells, are extensively involved in various important physiological processes such as plant growth and development regulation and adaptive responses to biotic/abiotic stresses [1]. FAs can be classified into two major categories based on the presence and number of double bonds in their hydrocarbon chains: saturated fatty acids (SFAs) and unsaturated fatty acids (UFAs) [2]. The biosynthesis of UFAs is primarily controlled by the fatty acid desaturase (FAD) family. These enzymes catalyze the formation of cis-double bonds at specific positions (e.g., Δ9 and Δ12 site) on fatty acyl chains, driving the conversion from monounsaturated to polyunsaturated states. As rate-limiting enzymes in fatty acid desaturation, FADs dynamically regulate membrane lipid composition by precisely modulating the degree of lipid unsaturation [3]. Under low temperatures, FAD-mediated lipid unsaturation maintains membrane stability. Conversely, under heat/oxidative stress, regulated desaturation prevents lipid peroxidation [4].

Based on differences in cofactors and subcellular localization, FAD-dependent desaturases are categorized into membrane-bound types (ω-3 and ω-6 desaturases) and soluble types (Δ4, Δ6, and Δ9 desaturases). Membrane-bound FADs are further classified into four functional subfamilies: FAD4, FAD2/FAD6, FAD3/FAD7/FAD8, and ADS/SLD/DES [5]. FAB2/SAD is currently the only known soluble FAD localized to the plastid stroma [6]. It initiates the unsaturated fatty acid synthesis cascade by catalyzing the Δ9-specific dehydrogenation of stearoyl-ACP (C18:0), producing palmitoleic acid (C16:1Δ9) and oleic acid (C18:1Δ9) [7]. This reaction system is subsequently extended by endoplasmic reticulum-localized Δ12 FADs, which convert oleic acid to linoleic acid (C18:2Δ9,12). This dienoic acid serves as a critical metabolic node, further modified by Δ15 FADs (ω-3 desaturases) or Δ6 FADs to generate α-linolenic acid and γ-linolenic acid, ultimately forming a complex network of unsaturated fatty acid metabolism [8].

FADs play a critical regulatory role in plant growth, development, and stress responses by modulating the synthesis and ratio of unsaturated fatty acids to maintain membrane fluidity and structural integrity, thereby enhancing plant stress tolerance [9]. For instance, overexpression of the cold-responsive soybean GmFAD3A gene significantly improves cold adaptation and germination traits in transgenic rice [10]. Conversely, upregulated expression of rice OsFAD7 and OsFAD8 reduces resistance to Phytophthora pathogens [11]. High expression of LeFAD3 in tomatoes is strongly correlated with enhanced photosynthetic capacity [12]. Under salt and alkali stress, HvFADs are notably involved in barley’s abiotic stress regulatory network [6]. Additionally, mitigating the interference of Foc TR4 on fatty acid synthesis mediated by the MaFAD gene can enhance banana resistance to fusarium wilt [13]. While FAD family members contribute to plant stress resistance, their individual roles in biotic vs. abiotic stress responses require clarification.

*Pyrus bretschneideri*, as one of the globally significant economic species, frequently faces abiotic stress challenges such as drought during cultivation, which severely inhibits growth and development and reduces fruit yield [14]. While studies have reported the identification of the pear GSK3 (glycogen synthase kinase 3) gene family and its role in drought stress response [15], the expression patterns of FAD genes in response to drought stress in pear remain unexplored. Therefore, this study aimed to conduct a comprehensive bioinformatics analysis of the FAD gene family in pear, leveraging genomic data to investigate protein physicochemical properties, conserved motifs, subcellular localization predictions, and gene structures. Additionally, the expression profiles of pear FAD genes under drought stress were analyzed. This research lays a foundation for future functional characterization of key drought-resistant candidate genes in pear.

## 2. Materials and Methods

### 2.1. Identification of the PbrFAD3 Gene Family in Pears

The pear genome annotation files, protein sequences, and nucleotide sequence data were downloaded from GigaDB (http://gigadb.org/ accessed on 20 March 2025). Hidden Markov models (HMMs) corresponding to the FA_Desaturase (PF00487), FA_Desaturase 2 (PF03405), and TMEM189 (PF10520) domains were obtained from the Pfam protein family database (http://pfam.xfam.org/ accessed on 20 March 2025). Using the HMM Search function in TBtools-IIv2.326, all potential FAD protein sequences were retrieved from the pear protein database [16], These candidate sequences were further validated using the SMART database (https://smart.embl.de/ accessed on 20 March 2025) to confirm their FAD protein identity.

### 2.2. Protein Characterization and Sequences Analyses

The physicochemical properties of pear PbrFADs, including amino acid number, molecular weight, isoelectric point (pI), and instability index, were analyzed using the TBtools-IIv2.326 software [17]. Subcellular localization prediction was performed using the WOLF PSORT online tool (https://wolfpsort.hgc.jp/ accessed on 20 March 2025).

### 2.3. Conserved Motif, Gene Structure, and Promoter Cis-Acting Element Analysis

In this study, the conserved motifs of pear FAD proteins were predicted using the MEME online tool (http://meme-suite.org/tools/meme accessed on 20 March 2025), with the number of motifs set to 20 [18]. Based on the analysis results and the gene-annotated GFF file, TBtools-IIv2.326 software was employed to integrate the conserved motifs with gene structure information, thereby generating a comprehensive visualization of the conserved motifs and gene structure of pear PbrFADs.

The promoter sequences (2000 bp upstream) of *PbrFADs* genes were extracted from the pear genome GFF annotation file using TBtools-IIv2.326. Subsequently, cis-acting elements in the promoters were analyzed using PlantCARE (http://bioinformatics.psb.ugent.be/webtools/plantcare/html/ accessed on 22 March 2025), and the results were visualized with TB tools software.

### 2.4. Chromosomal Localization Analysis, Synteny Analysis, and Protein–Protein Interaction Prediction

The chromosomal distribution of *PbrFADs* gene family members was visualized using TBtools-IIv2.326 software. Subsequently, TBtools was employed to identify relevant duplicated gene pairs, and the Advanced Circos function of TBtools was utilized to generate a visualization based on protein sequence similarity. Protein–protein interaction networks of PbrFADs were analyzed using the Arabidopsis protein interaction database (http://string-db.org/ 25 March 2025).

### 2.5. Phylogenetic Tree Construction

The protein sequences of five gene families from *Pyrus bretschneideri*, *Arabidopsis thaliana*, *Oryza sativa*, *Glycine max*, and *Triticum aestivum* were subjected to multiple sequence alignment using MEGA software (MEGA 11.0.13). A phylogenetic tree was constructed using the maximum likelihood (ML) method, followed by visual refinement and annotation of the resulting tree with the iTOL platform (https://itol.embl.de/ accessed on 20 March 2025).

### 2.6. Plant Materials

The experiment was conducted in May 2025 at the Handan City Bureau of Landscape and Forestry, China. The test material was *Pyrus bretschneideri*, sampled from the same location. Three uniformly growing pear seedlings were selected for drought stress treatment, with untreated plants serving as controls. Leaf samples were collected at 3, 6, 12, 24, and 48 h after drought stress application. For each time point, three independent biological replicates (leaves from different plants) were collected, immediately flash-frozen in liquid nitrogen, ground to fine powder, and stored in 2 mL cryotubes at −80 °C. Total RNA was extracted using the FastPure Universal Plant Total RNA Isolation Kit (Vazyme Biotech Co., Ltd., Nanjing, China). cDNA synthesis was performed with the HiScript III 1st Strand cDNA Synthesis Kit (+gDNA wiper) (Vazyme Biotech Co., Ltd., Nanjing, China).

### 2.7. Quantitative Expression Analysis by RT-qPCR

Gene-specific primers for *PbrFADs* were designed using Primer-BLAST (https://www.ncbi.nlm.nih.gov/tools/primer-blast accessed on 7 May 2025), with sequences listed in Table 1. Quantitative real-time PCR (qPCR) was performed using cDNA templates from pear samples collected at different time points and temperatures to analyze *PbrFAD* gene expression under drought stress. Each 20 µL reaction contained 10 µL of 2× Taq Pro Universal SYBR qPCR Master Mix (Vazyme Biotech Co., Ltd., Nanjing, China), 0.5 µL each of forward and reverse primers, 1 µL cDNA, and 8 µL ddH_2_O. The thermal profile included initial denaturation at 95 °C for 30 s, followed by 40 cycles of 95 °C for 5 s and 55 °C for 34 s. Relative expression levels were calculated via the 2−ΔΔCT method with three biological and technical replicates. Statistical analyses were performed using SPSS 22.0 software, while data visualization was generated using Excel 2013, TBtools-IIv2.326, and Graphpad Prism 10.1.2.

## 3. Results

### 3.1. Physicochemical Properties and Subcellular Localization Prediction of Pear PbrFAD Gene Family Members

Bioinformatic analysis of *PbrFAD* proteins are shown in Table 2. The pear *FAD* gene family members encode proteins ranging from 268 to 453 amino acids in length, with molecular weights varying from 30,598.65 Da (*PbrFAD27*, the smallest) to 51,589.82 Da (*PbrFAD7*, the largest). The predicted isoelectric points (pI) ranged from 5.41 (*PbrFAD3*) to 9.67 (*PbrFAD18*), with 28 members exhibiting basic properties (pI > 7). The instability indices varied between 27.84 (*PbrFAD23*) and 51.62 (*PbrFAD28*), indicating most PbrFADs are stable proteins (instability index < 40). Hydrophobicity analysis showed 23 PbrFADs had negative average GRAVY values, suggesting hydrophobic characteristics. Subcellular localization predictions identified 16 members in plastids, 2 in cytoplasm, 3 in endoplasmic reticulum, 10 in chloroplasts, and 1 each in cytoskeleton, mitochondria, and vacuole.

### 3.2. Phylogenetic Analysis of the Pear PbrFAD Gene Family

**Figure 1:**
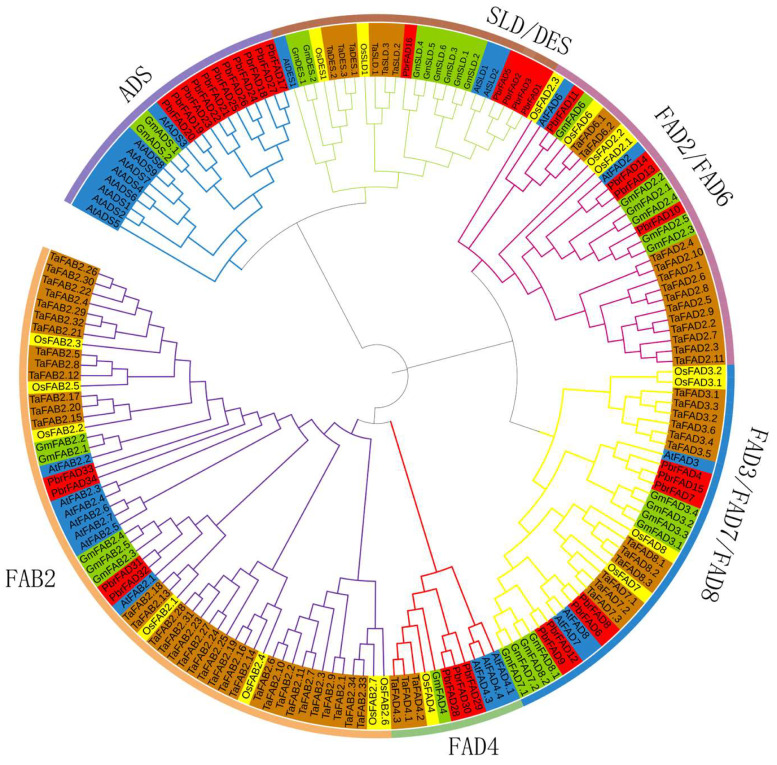
Phylogenetic analysis of FAD proteins in *Pyrus bretschneideri*, *Oryza sativa*, *Arabidopsis thaliana*, *Glycine max*, and *Triticum aestivum* revealed their classification into six distinct subfamilies: ADS, FAD4, FAB2, FAD3/FAD7/FAD8, FAD2/FAD6, and SLD/DES. In the phylogenetic tree, protein orthologs are color-coded by species: AtFAD (blue), PbrFAD (red), OsFAD (yellow), GmFAD (green), and TaFAD (brown).

To investigate the evolutionary relationships of the *PbrFAD* gene family, we performed phylogenetic analysis using FAD amino acid sequences from *Arabidopsis thaliana*, *Pyrus bretschneideri*, *Oryza sativa*, *Triticum aestivum*, and *Glycine max*. The resulting phylogenetic tree (Figure 1) revealed that the 34 *PbrFAD* genes could be classified into six distinct subfamilies: ADS, FAD4, FAB2, FAD3/FAD7/FAD8, FAD2/FAD6, and SLD/DES. Notably, the FAD4 subfamily contained 3 members, while the ADS subfamily was the largest with 11 members. The other subfamilies varied in size: FAD2/FAD6 (four members), FAD3/FAD7/FAD8 (seven members), SLD/DES (five members), and FAB2 (four members). Evolutionary analysis demonstrated that most PbrFAD subfamily members showed closer phylogenetic relationships with *Arabidopsis* and *Glycine max* than with *Oryza sativa* and *Triticum aestivum*, indicating functional evolution in dicots.

### 3.3. Motif and Gene Structure Analysis of the Pear PbrFAD Family

**Figure 2:**
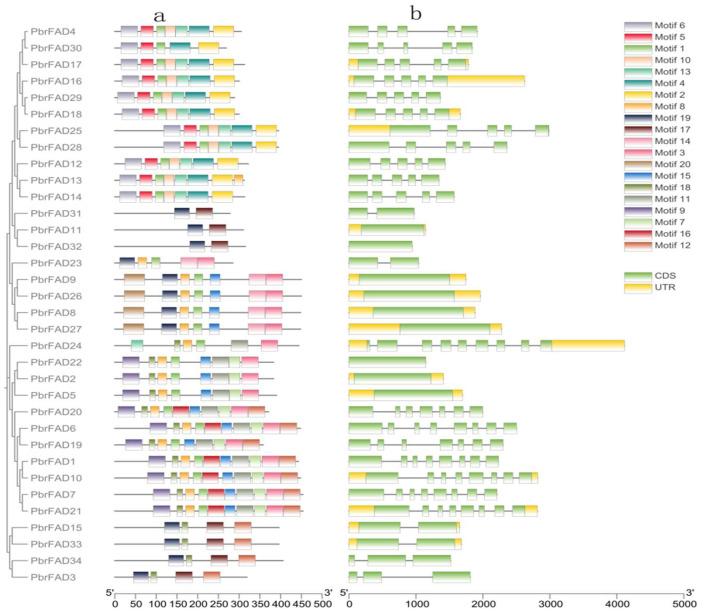
(**a**) Conserved motifs of the *PbrFAD* genes. The MEME URL was used for the motifs analysis of the 34 PbrFAD protein sequences. Colors of boxes represent motifs at the corresponding position in each PbrFAD protein; (**b**) gene structures of *PbrFAD* genes. Intron/exon structure of *PbrFAD* genes was analyzed by TBtools. Green boxes present exons, and single lines present introns. Gene models are drawn to scale, as shown in the bar at the bottom.

Structural characterization of the pear *FAD* gene family revealed both conserved patterns and distinct variations in domain organization and gene architecture across different subfamilies. Domain prediction analysis demonstrated that most FAD proteins contain motif1, motif3, and motif8, with motif1 and motif8 predominantly enriched in the C-terminal regions (Figure 2a).

Gene structure analysis further revealed that all PbrFADs possess complete coding sequences yet exhibit remarkable diversity in gene length and exon-intron organization. The number of exons varied from 1 to 9, while introns ranged from 0 to 8. Notably, *PbrFAD24* displayed the most complex architecture (nine exons/eight introns), whereas nine members (*PbrFAD11*, *PbrFAD32*, *PbrFAD9*, *PbrFAD26*, *PbrFAD8*, *PbrFAD27*, *PbrFAD22*, *PbrFAD2*, and *PbrFAD5*) contained only single exons. The number of introns and exons in other family members shows inconsistent patterns (Figure 2b).

### 3.4. Analysis of Cis-Acting Elements in the Promoters of Pear PbrFAD Genes

**Figure 3:**
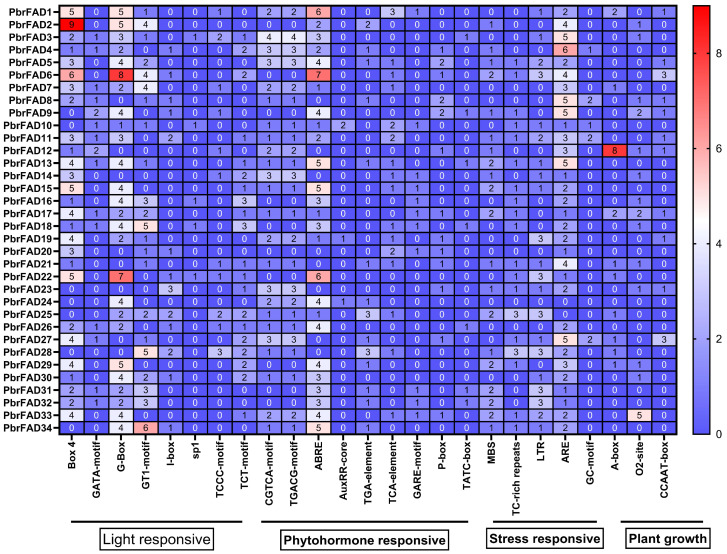
Analysis of promoter cis-acting elements of the pear PbrFAD gene family. Promoter element prediction analysis of the PbrFAD genes. Transcription factor binding sites within a region 2000 bp upstream of the PbrFADs start site was predicted. The number of putative TF sites in the promoters of the PbrFAD genes is marked.

Cis-regulatory element analysis of the 2000 bp promoter regions from 34 pear *PbrFAD* genes identified four major functional categories: light-responsive, hormone-responsive, stress-responsive, and plant growth/development-related elements. Light-responsive elements were most abundant (292 total), with G-box motifs being predominant (93 occurrences) though notably absent in the promoters of *PbrFAD8*, *PbrFAD12*, *PbrFAD14*, *PbrFAD20*, *PbrFAD21*, *PbrFAD23*, *PbrFAD27,* and *PbrFAD28*. Among hormone-responsive elements (251 total), abscisic acid (ABA)-related ABRE motifs were most frequent (87 occurrences). Stress-responsive elements (184 total) included drought-inducible MBS elements present in 20 PbrFAD promoters. The plant growth and development category (54 elements total) comprised 15 elements associated with meristem expression, 20 elements involved in zein metabolism regulation, and 3 elements related to circadian control (Figure 3).

### 3.5. Chromosomal Localization of the Pear PbrFAD Gene Family

**Figure 4:**
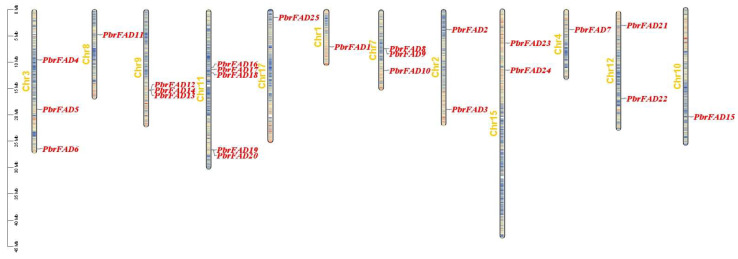
Distribution of *PbrFAD* genes on 12 of the 17 chromosomes in the Pyrus bretschneideri genome. The physical location map was drawn based on the location of the *PbrFAD* genes on the chromosomes by TBtools.

**Figure 5:**
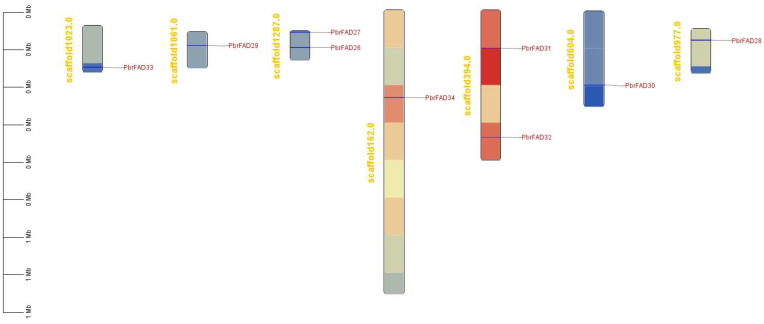
Nine *FAD* genes localized to the scaffold.

The 34 *PbrFAD* gene family members were distributed across 12 of 17 chromosomes, with 25 FAD genes localized to the chromosome (Figure 4) and 9 FAD genes localized to the scaffold (Figure 5). Chromosome 11 contained the highest number of members (five genes), while chromosomes 1, 4, 8, 10, and 17 each carried only a single member. Chromosomes 2, 12, and 15 harbored two members each, whereas chromosomes 3, 7, and 9 contained three members each. Notably, tandem duplication events were identified in three clusters: *PbFAD8* and *PbFAD9* on chromosome 7; *PbFAD12*, *PbFAD13*, and *PbFAD14* on chromosome 9; and *PbFAD19* and *PbFAD20* on chromosome 11.

### 3.6. Comparative Genomic Analysis of Synteny in the Pear PbrFAD Gene Family

**Figure 6:**
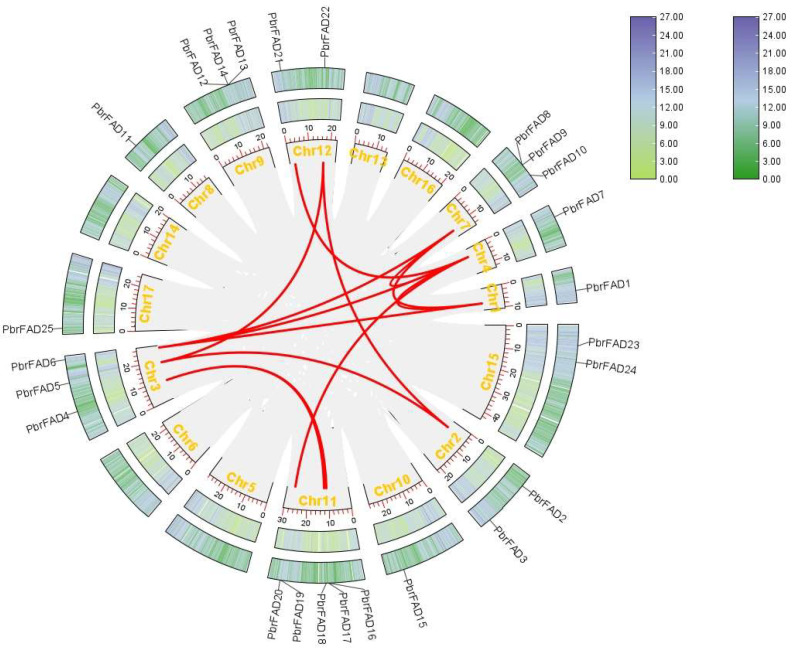
Collinearity analysis of the 34 identified *PbrFAD* genes. Collinearity analysis was performed using the TBtools software.

To investigate the evolutionary relationships of the pear *FAD* gene family, we performed collinearity analysis of *PbrFAD* genes within *Pyrus bretschneideri* and between *Pyrus bretschneideri* and *Arabidopsis thaliana*. Intra-species synteny analysis revealed 13 segmental duplication pairs among the 34 PbrFAD genes. Notably, *PbrFAD4* showed collinearity with both PbrFAD17 and PbrFAD18, while nine other genes (*PbrFAD5*, *PbrFAD2*, *PbrFAD21 PbrFAD22*, *PbrFAD7*, *PbrFAD6*, *PbrFAD10*, *PbrFAD1*, and *PbrFAD20*) each exhibited syntenic relationships with additional family members. These findings suggest lineage-specific expansion through segmental duplications, contributing to functional diversification of the *FAD* gene family in pear (Figure 6).

**Figure 7:**
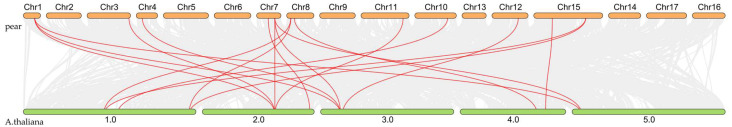
Interspecific collinearity analysis of *FAD* gene family members between pear and *A. thaliana* revealed 18 collinear relationships connecting 34 pear *FAD* genes with 27 *Arabidopsis* FAD orthologs.

Inter-species synteny analysis between pear and *Arabidopsis* revealed 18 collinear relationships connecting 34 pear *FAD* genes with 27 *Arabidopsis* FAD orthologs (Figure 7), demonstrating evolutionary conservation of genomic organization between these species.

### 3.7. Protein–Protein Interaction Network Analysis of Pear PbrFADs

**Figure 8:**
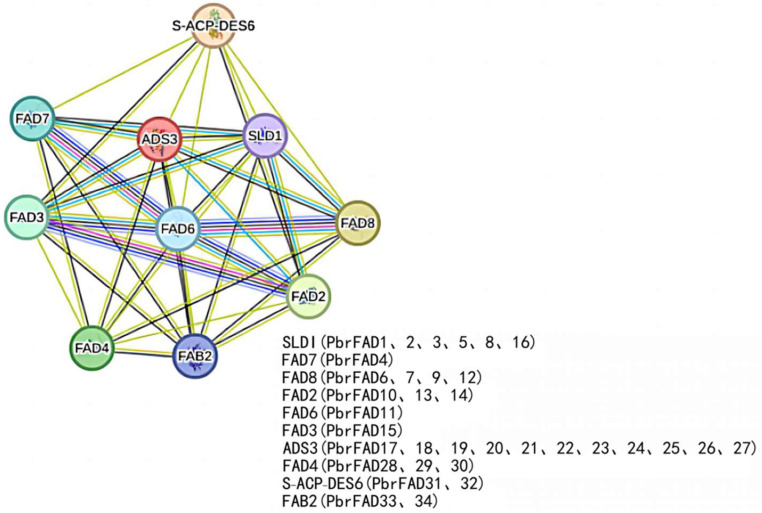
The protein–protein interaction network for *PbrFADs* based on their orthologs in Arabidopsis.

The protein interaction network revealed significant functional associations among *PbrFAD* members, with particularly strong interactions observed between FAD6, FAD7 and FAD8 (highest confidence scores) (Figure 8). Notably, four pear desaturases—*PbrFAD6*, *PbrFAD7*, *PbrFAD9*, and *PbrFAD12*—were identified as orthologs of *Arabidopsis AtFAD8*, all sharing conserved cold-inducible functions [19]; *PbrFAD10*, *PbrFAD13*, and *PbrFAD14* were identified as orthologous proteins to *Arabidopsis AtFAD2*, which is functionally involved in multiple stress responses, including abscisic acid (ABA) and salicylic acid (SA) signaling pathways as well as low-temperature and salt stress adaptation [20]; *Arabidopsis AtFAD6*, which is orthologous to pear *PbrFAD11*, has been demonstrated to be essential for salt tolerance during early seed growth and development [21]. In defense pathways, both *PbrFAD33* and its *Arabidopsis* ortholog FAB2 (along with *PbrFAD34*) play regulatory roles in salicylic acid (SA)- and jasmonic acid (JA)-mediated signaling [22]. The ADS3 clade, represented by pear *PbrFAD17* through *PbrFAD27*, is required for chloroplast biosynthesis under low-temperature conditions. Additionally, several *Arabidopsis* desaturases, including SLD1, FAD7, FAD3, S-ACP-DES6, and FAD4, are involved in the biosynthesis of key lipids such as palmitic acid, oleic acid, and glycosylceramides, highlighting the conserved yet diversified functions of *FAD* family proteins across species.

### 3.8. Quantitative Real-Time PCR (qRT-PCR) Analysis of Pear PbrFAD Gene Family Expression Patterns

**Figure 9:**
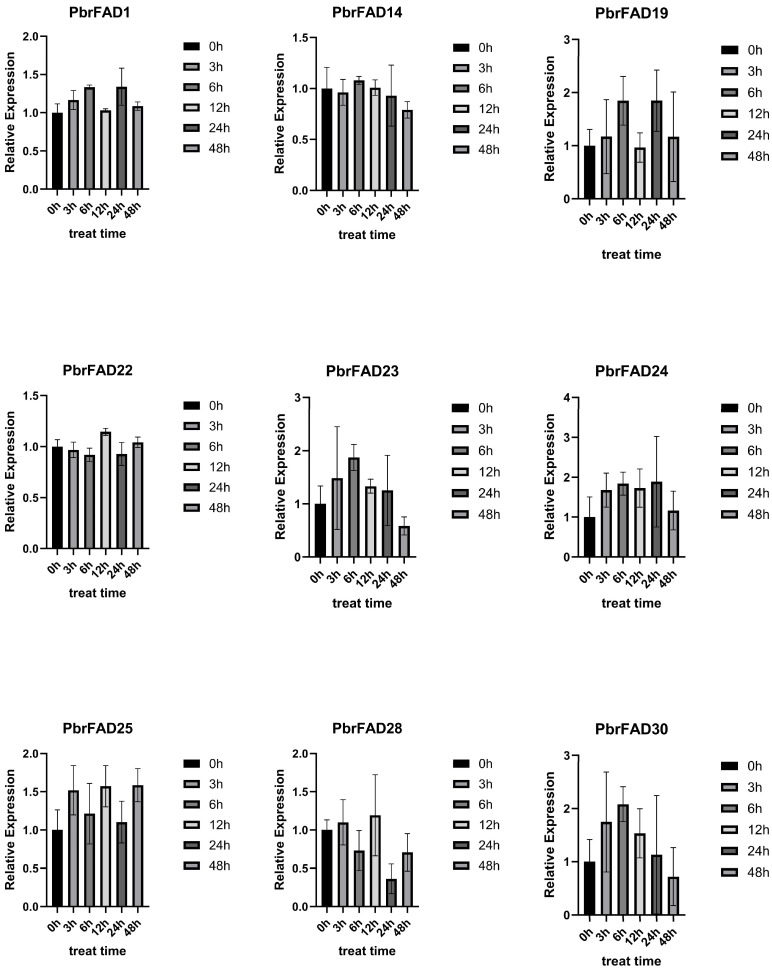
Quantitative Real-Time PCR Analysis of the *PbrFADs* Gene Family in Pear.

This study employed qRT-PCR to analyze drought-responsive expression patterns of *PbrFAD* genes (Figure 9). Based on pear transcriptome data under drought stress, nine significantly upregulated genes (*PbrFAD1*, *PbrFAD14*, *PbrFAD19*, *PbrFAD22*, *PbrFAD23*, *PbrFAD24*, *PbrFAD25*, *PbrFAD28*, and *PbrFAD30*) were selected for validation. Distinct temporal expression profiles were observed: *PbrFAD1*, *PbrFAD19,* and *PbrFAD24* showed initial upregulation (0–6 h), subsequent downregulation (6–12 h), followed by secondary induction (12–24 h), and final repression (28–48 h). *PbrFAD23* and *PbrFAD30* exhibited rapid induction (0–6 h) before declining. Notably, *PbrFAD22* and *PbrFAD24* displayed opposing expression trends. Complex oscillation patterns were seen in *PbrFAD25* and *PbrFAD28* with alternating induction (0–3 h; 6–12 h) and repression phases (3–6 h; 12–48 h). *PbrFAD14* demonstrated unique dynamics with initial downregulation (0–3 h), transient recovery (3–6 h), and progressive suppression thereafter. These results demonstrate that all nine *PbrFAD* genes respond differentially to varying durations of drought stress, revealing intricate temporal regulation mechanisms.

## 4. Discussion

The fatty acid desaturase (FAD) gene family, as core regulators of plant membrane lipid metabolism and stress response, has been systematically identified in various plant species [23]. For instance, in monocot plants, the rice (*Oryza sativa*) genome contains 20 *FAD* genes [5]; the wheat (*Triticum aestivum*)genome contains 68 FAD genes [24]. Among dicotyledon plants, 84 FAD genes have been identified in rapeseed (*Brassica napus*) [25]; the *Arabidopsis thaliana* and cucumber (*Cucumis sativus*) [26] genomes each contain 23 *FAD* genes; and 29 *FAD* genes have been characterized in soybean (*Glycine max*) [10]. In this study, we report the first identification of 34 PbrFADs in the pear (*Pyrus bretschneideri*) genome. This number is significantly higher than that in the monocot model plant *Oryza sativa* (20 genes) yet comparable to that in the dicot model *Arabidopsis thaliana* (27 genes). Furthermore, phylogenetic analysis revealed that pear shares a closer evolutionary relationship with *Arabidopsis thaliana* than with *Oryza sativa*. This divergence likely stems from their shared classification as dicotyledonous plants, whereas rice belongs to the monocot clade [27]. Evolutionary analysis of the six subfamilies (ADS, FAD4, FAB2, SLD/DES, FAD2/FAD6, and FAD3/FAD7/FAD8) revealed distinct patterns of gene family expansion and conservation. The ADS subfamily showed significant expansion in pear with 11 members while being completely absent in monocots such as banana [13] and barley (*Hordeum vulgare*) [6], suggesting its emergence after the divergence of monocot and dicot lineages. Notably, all members of the FAD4 and SLD/DES subfamilies exhibited identical conserved motifs and exon–intron structures, indicating high sequence conservation and close phylogenetic relationships within these subfamilies. In contrast, substantial structural divergence was observed among other subfamilies, reflecting functional diversification during evolution.

Genome-wide analysis identified 34 *PbrFAD* genes unevenly distributed across 12 chromosomes (1, 2, 3, 4, 7, 8, 9, 10, 11, 12, 15, and 17) in pear (*Pyrus bretschneideri*), with chromosomes 3, 7, and 9 each harboring three members and nine *FAD* genes localized to the scaffold. Notably, chromosome 11 exhibited significant regional enrichment with five *PbrFAD* genes. Compared to the reported FAD family size in other Rosaceae species (e.g., 68 in strawberry [28]), the relatively compact *PbrFAD* gene repertoire suggests potential lineage-specific gene loss or functional consolidation events during pear genome evolution.

Gene duplication has played an indispensable role in the evolutionary process as a key driver of functional diversification within the *FAD* gene family [29]. Comparative genomic analysis revealed that the expansion of the *FAD* gene family in pear (*Pyrus bretschneideri*) involves 4 tandem and 13 segmental duplication events. Similarly, the Brassica napus FAD family contains 3 tandem and 25 segmental duplicates [25], while wheat (*Triticum aestivum*) exhibits 26 tandem and 126 segmental duplicates [24], collectively suggesting that segmental duplications represent the predominant mechanism for FAD family expansion. Furthermore, interspecies synteny analysis between pear and *Arabidopsis thaliana* demonstrated substantial conservation of homologous genes, indicating shared evolutionary trajectories among dicot species.

Promoter analysis of PbrFADs has elucidated the molecular basis for their responsiveness to complex environmental signals, revealing that transcription factors play pivotal roles in coordinating signaling cascades and abiotic stress responses [30]. Functionally, these regulatory molecules bind to promoter regions of target genes to either activate or suppress their expression [31]. Systematic examination of the pear *FAD* gene family identified an abundance of cis-acting elements associated with growth, development, and stress responses, including ABRE, MBS, LTR, A-box, and GATA-motif elements within their promoter regions. Previous studies have demonstrated that these elements mediate transcriptional responses to various abiotic stresses (e.g., salinity, drought, and cold) through specific interactions with transcription factors [31]. Among these, MBS elements were enriched in 20 genes, TCA-elements were detected in 16 genes, and low-temperature responsive elements were distributed across 21 genes. Notably, antioxidant response elements represented the most prevalent regulatory motif in *PbrFADs.* The co-occurrence of drought-responsive and antioxidant elements suggests a synergistic mechanism whereby functional complementarity, signal integration, and spatiotemporal coordination collectively enhance plant adaptive capacity under drought stress, providing a promising molecular strategy for improving crop stress tolerance.

Expression profiling via qRT-PCR revealed distinct drought-responsive patterns among nine *PbrFAD* genes in pear, with *PbrFAD23* and *PbrFAD30* exhibiting significant upregulation under drought stress followed by temporal decline, suggesting their pivotal roles in drought adaptation. These findings align with prior reports demonstrating enhanced drought tolerance mediated by NtFAD3/NtFAD8 in tobacco [32], salt-responsive BnFAD7.4 in rapeseed, and stress-inducible MdFAD11/28 in apple, thereby providing robust cross-species validation of *FAD* genes’ conserved functions in abiotic stress responses.

## 5. Conclusions

Through comprehensive bioinformatics analysis, we identified 34 fatty acid desaturase (FAD) genes in the pear (*Pyrus bretschneideri*) genome. Phylogenetic reconstruction classified these PbrFAD proteins into six distinct subfamilies: FAD4, FAB2, ADS, SLD/DES, FAD2/FAD6, and FAD3/FAD7/FAD8. Chromosomal localization revealed uneven distribution across 12 pear chromosomes, with family expansion likely driven by segmental duplication events. Cis-element analysis of *PbrFAD* promoters indicated potential complex regulatory mechanisms, particularly through stress-responsive elements. qRT-PCR validation demonstrated that *PbrFAD23* and *PbrFAD30* were significantly upregulated during early drought stress (0–6 h), suggesting their crucial roles in pear’s drought adaptation response.

## Figures and Tables

**Table 1:** Real-time quantitative PCR primers.

Name	Forward Primers (5′-3′)	Reverse Primers (5′-3′)
*Actin*	GCGGTTATGCCCTCCCTC	CGATTTCCCGTTCAGCAGTAG
*PbrFAD1*	ACGCGCACCAACATCCAATA	TCCCTCCTCCACGTCCAAAT
*PbrFAD14*	AATGGCCCAGTGGGGTTATG	CCGGACCCATTTTGACTTCC
*PbrFAD19*	ACGGATCATTGCAAGCGGAT	CAGCTGTGGGTTACTTGTCG
*PbrFAD22*	GCCATGCGGCTCGTTTATTT	TTTCAACCCAAAAGCCGCC
*PbrFAD23*	CACCCACAAAGCCTGGAGAT	TGAAGAACTGGTCGAGGTGC
*PbrFAD24*	ACGTCTGTCTGTTTCCTAGCG	ATCTGTCACTCGTCGCTCTTC
*PbrFAD25*	TTGCGGCGAACTATGGAAGT	AGCTAAAGCAGGCAAGGTGT
*PbrFAD28*	TTGCGGCGAACTATGGAAGT	ATCCCCGACCCTGTCCATTA
*PbrFAD30*	CCTGCCGACACACCTTTAGT	GTTCGAAAACCCTTCTCGGC

**Table 2:** Physical and Chemical Properties Analysis and Subcellular Localization of *PbrFAD* Gene Family Members in Pear.

Gene Name	Sequence ID	Number of Amino Acid	Molecular Weight	Theoretical pI	Instability Index	Aliphatic Index	Grand Average of Hydropathicity	Subcellular Localization
*PbrFAD1*	*Pbr009536.1*	449	51,350.4	8.88	34.81	94.45	0.037	Plas
*PbrFAD2*	*Pbr019246.1*	447	51,283.19	8.51	41.77	89.19	0.002	Plas
*PbrFAD3*	*Pbr041422.1*	449	51,416.48	8.88	34.48	92.94	0.03	Plas
*PbrFAD4*	*Pbr034778.1*	370	42,904.29	8.73	32.82	84.84	−0.182	E.R.
*PbrFAD5*	*Pbr000682.1*	447	51,253.17	8.65	41.67	89.19	−0.006	Plas
*PbrFAD6*	*Pbr042189.1*	442	50,222.47	8.96	38.37	81.33	−0.282	Chlo
*PbrFAD7*	*Pbr021630.1*	447	51,589.82	7.43	38.84	78.26	−0.293	Chlo
*PbrFAD8*	*Pbr029449.1*	387	44,507.52	8.93	42.43	81.32	0.077	Chlo
*PbrFAD9*	*Pbr029451.1*	453	51,523.93	8.93	39.21	81.7	−0.268	Plas
*PbrFAD10*	*Pbr010914.1*	389	44,907.75	8.95	44.23	87.89	−0.067	E.R.
*PbrFAD11*	*Pbr004162.1*	443	50,758.89	9.12	42.77	83.93	−0.136	Chlo
*PbrFAD12*	*Pbr005175.1*	453	51,449.74	8.75	39.39	80.18	−0.268	Plas
*PbrFAD13*	*Pbr005172.1*	382	43,897.72	8.9	37.04	86.02	−0.035	Plas
*PbrFAD14*	*Pbr005174.1*	382	43,828.48	8.46	38.76	87.02	−0.026	Pero
*PbrFAD15*	*Pbr039381.1*	357	41,306.13	8.27	31.79	80	−0.336	Chlo
*PbrFAD16*	*Pbr029387.1*	284	32,206.16	9.06	44.79	87.15	0.095	Vacu
*PbrFAD17*	*Pbr005597.1*	304	35,002.43	9.58	39.05	91.71	0.024	Chlo
*PbrFAD18*	*Pbr008511.1*	312	35,894.55	9.67	34.78	89.26	−0.01	Cyto
*PbrFAD19*	*Pbr033732.1*	394	45,209.18	9.57	45.61	83.43	−0.131	Plas
*PbrFAD20*	*Pbr033731.1*	394	45,228.14	9.59	47.26	82.44	−0.132	Plas
*PbrFAD21*	*Pbr028873.1*	311	36,193.45	9.3	28.19	94.6	−0.059	Plas
*PbrFAD22*	*Pbr035830.1*	312	36,642.03	9.15	31.6	91.47	−0.097	Plas
*PbrFAD23*	*Pbr019851.1*	321	37,098.78	9.35	27.84	93.8	−0.068	Plas
*PbrFAD24*	*Pbr034249.1*	299	34,942.71	9.17	31.32	90.3	0.143	Plas
*PbrFAD25*	*Pbr005674.1*	288	33,425.63	9.1	32.82	83.61	0.036	Plas
*PbrFAD26*	*Pbr005593.1*	299	34,765.36	9.07	30.4	89.33	0.134	Plas
*PbrFAD27*	*Pbr005591.1*	268	30,598.65	9.36	34.61	99.29	0.219	Plas
*PbrFAD28*	*Pbr042237.1*	277	30,929.16	7.81	51.62	82.71	−0.265	Mito
*PbrFAD29*	*Pbr001965.1*	309	34,104.06	6.69	48.19	93.07	0.002	Chlo
*PbrFAD30*	*Pbr033270.2*	314	34,699.62	6.94	50.3	89.75	−0.091	E.R.
*PbrFAD31*	*Pbr024876.2*	395	44,487.69	5.93	38.77	80.33	−0.404	Chlo
*PbrFAD32*	*Pbr024899.1*	395	44,444.67	5.82	39.11	81.32	−0.383	Chlo
*PbrFAD33*	*Pbr001251.1*	405	46,358.82	6.32	38.14	84.07	−0.428	Chlo
*PbrFAD34*	*Pbr009762.1*	318	36,337.42	5.41	34.13	84.06	−0.397	Cyto

## Data Availability

The data presented in this study are available on request from the corresponding author. The data are not publicly available due to ethical reason.
